# Supporting Persons With Severe Dementia in Communicating Their Preferences

**DOI:** 10.1093/geroni/igaa057.1470

**Published:** 2020-12-16

**Authors:** Vanessa Burshnic, Michelle Bourgeois

**Affiliations:** 1 Durham VA Medical Center, Durham, North Carolina, United States; 2 University of South Florida, Tampa, Florida, United States

## Abstract

Government mandates require US nursing homes to provide preference-based, person-centered care. Persons with dementia (PWD) are less likely to have a role in preference assessments (PAs) used for care planning due to communication challenges associated with the disease. Thus, PWD are at risk of receiving de-personalized treatments. External supports (photograph and text cues) are known to improve communication in PWD. Yet these cues have never been studied with widely used PAs, such as the MDS 3.0 Section F and Preferences for Everyday Living Inventory (PELI). This study examined the effect of two PA conditions (externally supported; standard verbal) on preference consistency and response types (off-topic, clarification requests, elaboration) of residents with severe dementia (N=21) (BIMS < 7) when assessed twice, one-week apart. PA questions were derived from the MDS 3.0 Section F and PELI. As a social validity measure, naïve judges (N=10) listened to interviews and rated residents’ communication clarity and their confidence understanding residents’ preferences. Results showed that neither condition promoted significantly greater levels of consistency over time. Residents’ ‘clarification requests’ were significantly fewer with use of external supports. Other response types were not significantly different across conditions. Judges’ ratings were not significantly different across conditions; however, they rated residents’ communication as clear and understandable overall. This study addresses a gap in current research and holds important implications for helping providers meet government mandates and enhance care plan participation by residents with severe dementia and other communication challenges.

